# Severe hypoglycemic coma with radiological evidence of hypoglycemic encephalopathy due to impaired awareness to hypoglycemia: a case report

**DOI:** 10.3389/fmed.2026.1680929

**Published:** 2026-03-11

**Authors:** Abdulmalik M. Aloriney

**Affiliations:** Department of Family and Community Medicine, College of Medicine, Imam Mohammad Ibn Saud Islamic University (IMSIU), Riyadh, Saudi Arabia

**Keywords:** hypoglycemic encephalopathy, hypoglycemia, diabetes mellitus, coma, encephalopathy

## Abstract

Adults with diabetes are at a higher risk of recurrent hypoglycemia, a condition linked to morbidity and mortality. This is due to their diminished counter-regulatory response to hypoglycemia, which can lead to impaired awareness to hypoglycemia (IAH). The brain relies on circulating glucose as its primary energy source, so hypoglycemia impairs central nervous system function. Clinical manifestations vary depending on severity, duration, and serum glucose responsiveness to treatment. Hypoglycemic encephalopathy (HE) is characterized by coma or stupor in patients with glucose levels below 50 mg/dL, despite normalization. Early recognition of symptoms is crucial to prevent irreversible brain cell death and timely intervention to restore blood glucose levels and minimize complications. We report a case of a 42-year-old man with type 1 diabetes who presented in hypoglycemic coma (GCS 3). Neuroimaging confirmed hypoglycemic encephalopathy, and the patient improved after intravenous dextrose and supportive care. The current case showed that IAH causes severe sequelae due to the progression of the hypoglycemia affecting the brain and leading to impaired quality of life associated with bed-ridden outcomes and inability to work.

## 1 Introduction

Adults with diabetes mellitus who are treated with insulin or insulin secretagogues are at increased risk of recurrent hypoglycemia, a condition linked to considerable morbidity and mortality, due to their diminished counter-regulatory response to hypoglycemia, which can result in impaired awareness to hypoglycemia (IAH) following repeated episodes (1). The brain depends on circulating glucose as its primary energy source; thus, hypoglycemia endangers normal brain function. Clinically, hypoglycemia impairs central nervous system function, affecting anterior brain regions before more caudal regions (2). The clinical manifestations of hypoglycemia are complex and depend on severity, duration, and serum glucose responsiveness to initial treatment. Severe hypoglycemia is characterized by isoelectric electroencephalography (EEG) readings, cerebral cortical function falls while maintaining medullary activity confirmed by sustained respiratory and cardiovascular functions (3).

Hypoglycemic encephalopathy (HE) refers to diffuse brain dysfunction attributable to severe and often prolonged hypoglycemia. While cognitive and neurological dysfunction may emerge once plasma glucose falls to approximately 45–50 mg/dL, encephalopathy and coma typically occur with sustained and/or profound hypoglycemia, frequently < 30 mg/dL (4). When defining HE, it is also essential to consider and exclude other causes of encephalopathy, including hepatic or renal failure, chronic alcohol use, toxins, sepsis, electrolyte abnormalities, and hypoxic–ischemic injury. Neurological manifestations in hypoglycemic encephalopathy (HE) encompass aphasia, hemiplegia, hemianopsia, seizures, lethargy, and coma as hypoglycemia advances (5). Hypoglycemic encephalopathy manifests as confusion and delirium when blood glucose levels fall below 2.5 mmol/L (45 mg/dl). There is a faster drop in the cerebral metabolic rate of glucose (CMRglc) than cerebral metabolic rate of oxygen (CMRO2). This suggests that the brain is using other substrates, like tricarboxylic acid (TCA) cycle intermediates and amino acids (like glutamine and glutamate) (6). These substrates supply transient energy in the absence of glucose. When blood glucose levels decline below 2 mmol/L (36 mg/dl), EEG alterations manifest as increased amplitude and decreased frequency, subsequently followed by diminished amplitude and frequency approaching 1 mmol/L (18 mg/dl). When ATP levels in the brain fall below 1 mM, the EEG becomes isoelectric, resulting in coma (7). Awareness and early recognition of hypoglycemic encephalopathy are crucial to prevent irreversible brain cells death. Prompt recognition of symptoms, such as confusion, neurologic deficits, or altered EEG patterns, allows timely intervention to restore blood glucose levels and minimize complications.

## 2 Case presentation

**Figure 1:**
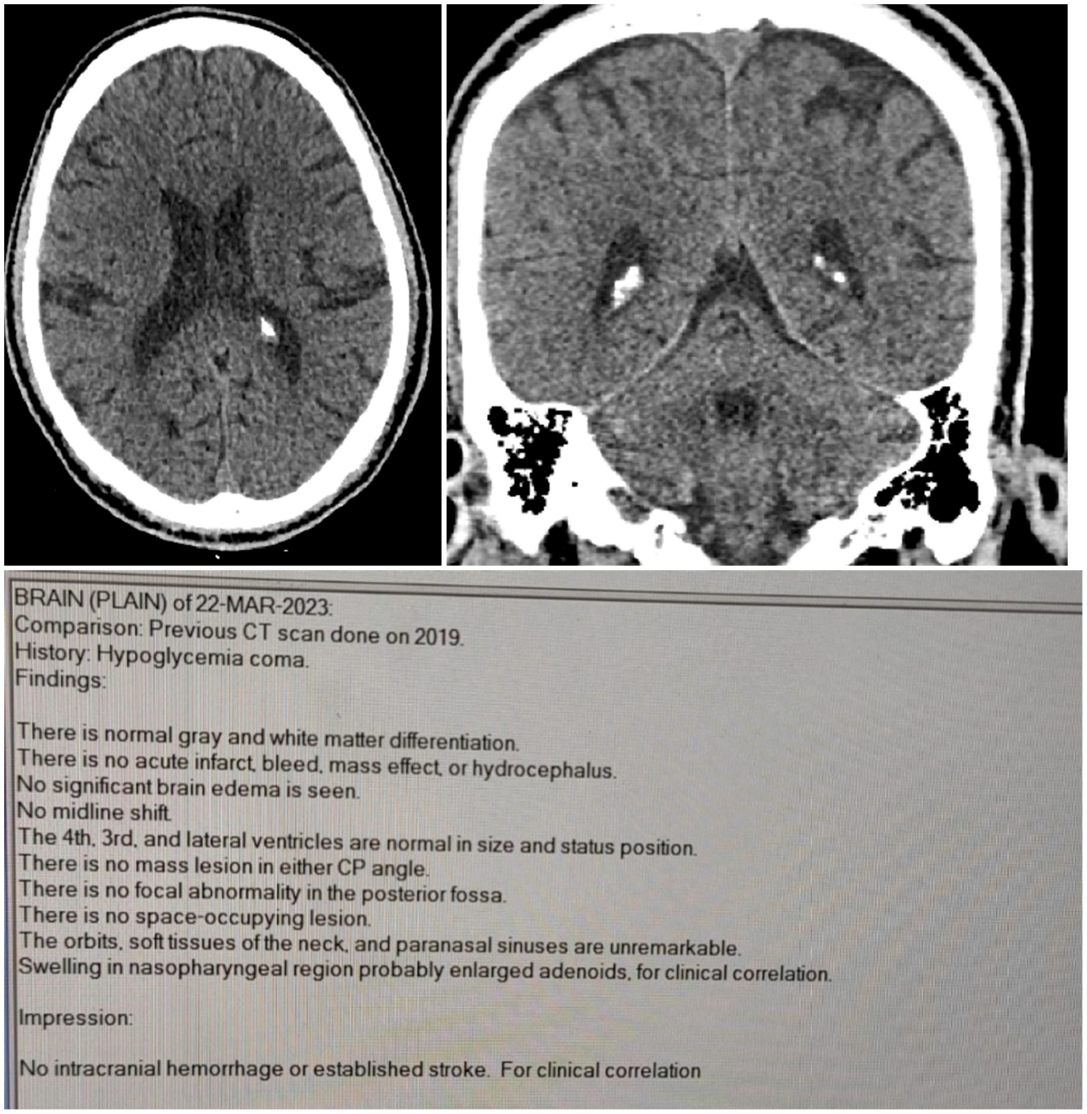
Non-contrast head CT. No acute intracranial hemorrhage, mass effect, or midline shift; ventricles normal in size and configuration.

**Figure 2:**
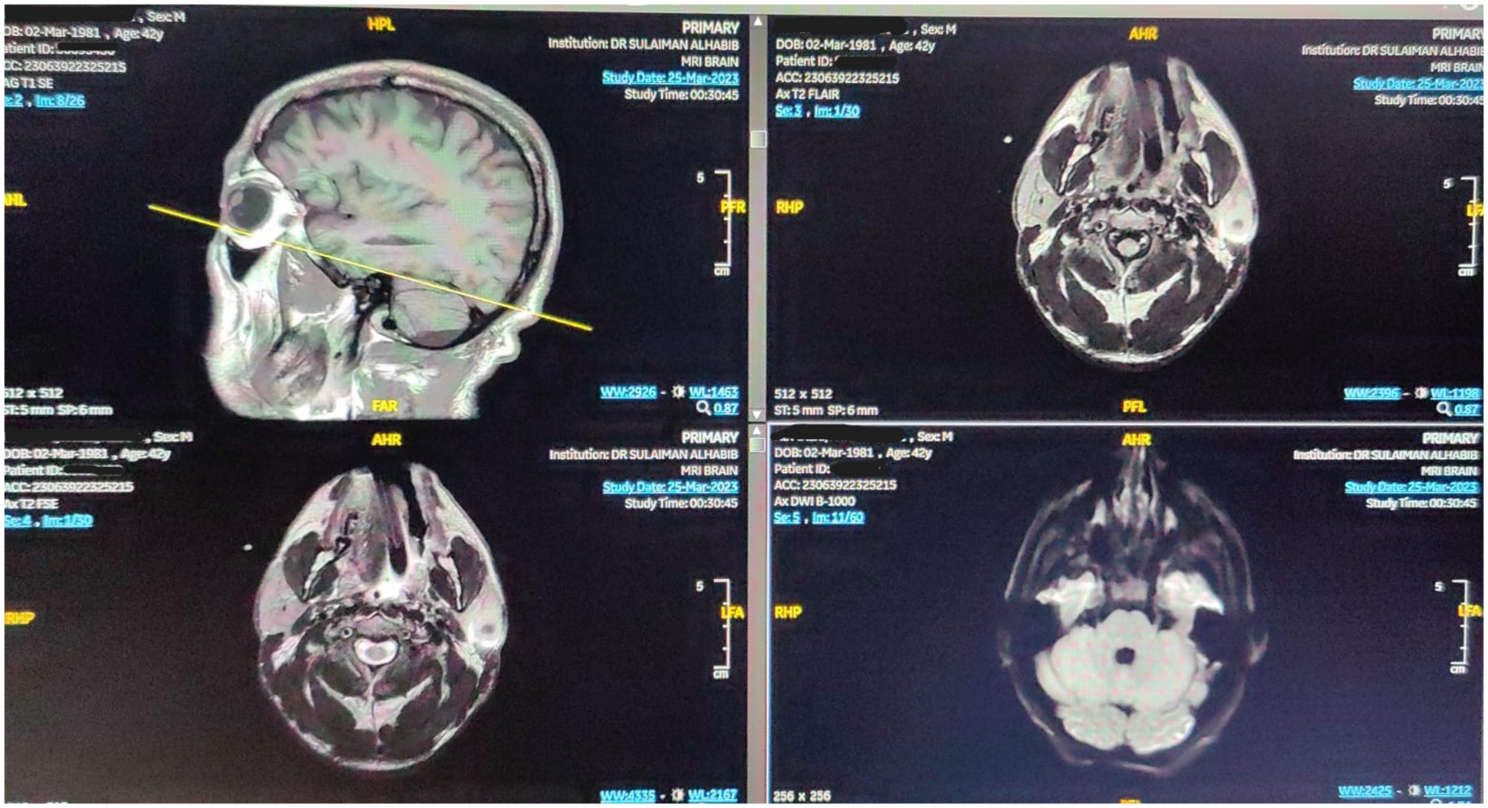
Non-contrast brain MRI. DWI shows cortical diffusion restriction predominantly in the parietal and parieto-occipital regions with corresponding low ADC; FLAIR shows cortical/subcortical hyperintensity.

**Figure 3:**
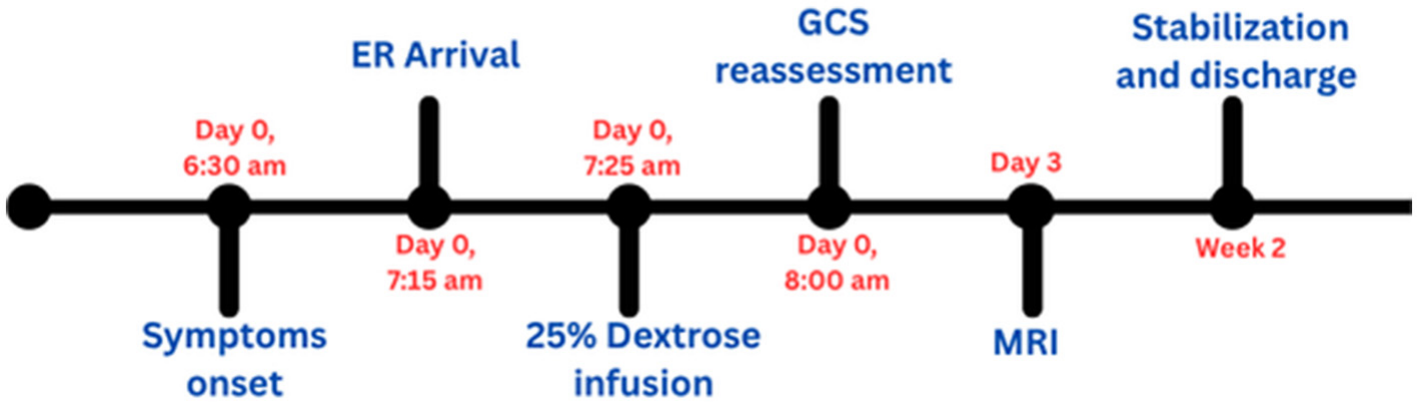
Timeline of clinical course and interventions.

A 42-year-old man who has longstanding type 1 diabetes mellitus was admitted to the emergency department. He was on a basal–bolus multiple daily injection regimen was admitted to the emergency department. His outpatient insulin regimen consisted of insulin glargine 50 units once daily as basal insulin and insulin aspart 30 units three times daily before meals as prandial insulin. The patient reported that he routinely administered these fixed doses with meals without adjusting the rapid-acting insulin for carbohydrate content or meal size. On admission, he was unresponsive to verbal commands and to painful stimulation; the pre-intubation Glasgow Coma Scale was E1V1M1. Endotracheal intubation was performed in the ER department and was placed on mechanical ventilation. The vital parameters have normalized later, and the initial blood glucose level was 18 mg/dL. The patient was given 25% dextrose intravenously over 10 min to restore serum glucose rapidly and prevent further neuronal injury, followed by 5% dextrose over 1 h and the hypoglycemia management protocol with supportive care (IV fluids and electrolyte management) was initiated. For close monitoring in the emergency room, cranial computed tomography (CT) was free and did not show acute intracranial hemorrhage or established stroke (Figure 1 shows CT report). A blood sample was drawn for laboratory investigations, which showed elevated liver (gamma GT, AST, ALT) and cardiac enzymes (NT-proBNP, troponin I), leukocytosis with lymphopenia, low bicarbonate and phosphorus. Laboratory findings of clinical importance are presented in Table 1 and all patients' labs are mentioned in Supplementary Tables S1, S2. Moreover, urine analysis and virology were free. Cerebral magnetic resonance imaging (MRI) without contrast was performed 72 h after the admission in which nasogastric tube (NGT) and endotracheal tube (ETT) were seen. Additionally, it showed changes that suggest hypoglycemic encephalopathy, such as bilateral parietal and parieto-occipital cortex and to a lesser extent bilateral basal ganglia abnormal signal intensity on diffusion-weighted imaging (DWI) and fluid-attenuated inversion recovery (FLAIR) (Figure 2). A diagnosis of hypoglycemic coma was made. Two weeks after admission, the initial neurological status of the patient stabilized with no further improvement, and he was discharged home with chronic home care preparation (Figure 3 illustrates timeline of clinical course and clinical interventions during patient hospitalization). However, he became bed-ridden and lost his job and this shows the bad sequalae and major complications associated with IAH which is a very common issue in diabetic patients.

**Table 1:** Showing important laboratory findings of the case.

**Description**	**Result value**	**Reference range**
HbA1c (Glycosylated)	6.90 %	
C-Peptide	< 0.03 ng/mL	1.11–4.43 ng/mL
NT-proBNP (N-term)	126.70 pg/mL	< 125 pg/mL
Troponin I HS	0.118 ng/L	< 0.028 ng/L
Bicarbonate (CO2)	17 mmol/L	22–29
AST/GOT	45 U/L	< 35 U/L
ALT/GPT	58 U/L	< 55 U/L
CRP high sensitivity	327.06 mg/L	< 10 mg/L
Phosphorus	0.66 mmol/L	0.74–1.52 mmol/L
WBCs	15.19 × 10^9^/L	4–11 × 10^9^/L
NEUTROPHILS	12.28 × 10^9^/L	2–6.9 × 10^9^/L
NEUT%	80.870	37–80
LYM%	8.81	10–50
MONOCYTES	1.439 × 10^9^/L	0.2–0.9 × 10^9^/L
Platelets	315 × 10^9^/L	150–450 × 10^9^/L
MPV	7.87 fL	7.4–10.4 fL
PT (Prothrombin Time)	12.90 s	
INR (International N.)	1.08	
PTT (Partial Thromb.)	28.60 s	25–33 s
D-DIMER	0.37 mg/L FEU	0–0.5 mg/L FEU

H, High; L, Low; N, Normal.

## 3 Discussion

Hypoglycemia, which is defined as prolonged low serum glucose levels, is the most common endocrinological emergency. It can clinically and radiologically mimic other neurological conditions, including ischemic stroke (8, 9). Severe, life-threatening hypoglycemia has a prevalence of once per year for patients with insulin-based diabetes mellitus. Brain glucose deprivation mediates the increased morbidity resulting from severe hypoglycemia, leading to focal and global neurological deficits such as hemiplegia, seizures, coma, and brain damage (10, 11). The mechanism by which hypoglycemia causes brain damage remains entirely unclear, but many theories try to explain it. The increased intracellular calcium influx due to excitotoxicity has been hypothesized to play an important role in hypoglycemia through brain cell necrosis (12). Another theory suggests that a decrease in serum glucose promotes cellular energy depletion in neurons and astrocytes, which in turn leads to the failure of membrane ionic pumps, impaired protein synthesis, excessive aspartate production, and loss of membrane ion homeostasis. This, in turn, initiates a shift of water from the extracellular space into the intracellular space (13, 14). This excitotoxic edema appears in diffusion-weighted imaging (DWI) as a hyperintense signal with a corresponding decreased apparent diffusion coefficient. The cerebral cortex, dorsal striatum, and hippocampus commonly experience hypoglycemia-induced excitotoxic edema, while the cerebellum, brain stem, or hypothalamus rarely experience it (15). Moreover, neurotransmitter dysregulation also plays a significant role in the physiopathology of hypoglycemia-related neuronal injury. The glucose oxidation produces precursors for neurotransmitters including glutamate and acetylcholine. Glutamate and glutamine serve as additional energy sources, contributing to intracellular alkalosis caused by ammonia and the accumulation of aspartate in the extracellular sector, potentially leading to selective neuronal necrosis (16).

We present a case of hypoglycemic encephalopathy. The laboratory blood tests showed significant hypoglycemia on admission. Diffusion-weighted brain MRI and FLAIR sequence revealed hyperintensities in the parietal and parieto-occipital cortex in both hemispheres and, to a lesser extent, bilateral basal ganglia abnormalities with no midline shift or other brain abnormalities. In addition, paranasal sinuses visualization demonstrated sphenoid and bilateral maxillary sinusitis. Serological tests and PCR were done to exclude possible infection and results were negative. These findings were attenuated on the first few days of admission after management of patients with live saving measures. MRI was conducted around the 3rd day of admission for follow up of brain condition. Based on clinical and radiological characteristics, this case was diagnosed as hypoglycemia induced brain injury evidenced by the reversibility of symptoms after early administration of glucose as described earlier.

## 4 Conclusion

Hypoglycemia is a serious condition characterized by low blood glucose levels with subsequent brain damage. Early detection of hypoglycemia, particularly in high-risk populations, is crucial to prevent the progression of encephalopathy and reverse the condition through early administration of glucose formulations. Cerebral neuroimaging and early measurement of serum glucose play a key role in distinguishing hypoglycemic encephalopathy from other conditions such as ischemic infarctions. IAH is a recognized complication among insulin-treated individuals with diabetes, and it is associated with major complications and bad sequalae, therefore, diabetic patients should be aware of this issue to overcome the occurrence of hypoglycemia and the association IAH which result in bed-ridden complications as the presented case. Moreover, Preventive strategies such as structured patient education, use of continuous glucose monitoring (CGM), individualized insulin dose adjustment, and routine screening for impaired awareness of hypoglycemia can help reduce the risk of recurrence in similar cases. Further research is required to better understand the potential mechanisms, optimize diagnostic approaches, and raise awareness about hypoglycemia and its consequences.

## 5 Patient consent

Written informed consent was obtained from the patient for publication of this case report and accompanying images. The patient was informed about the nature of the report, including the purpose of sharing their clinical information, and the potential risks and benefits of publication. The patient was assured that all efforts would be made to protect their privacy and maintain confidentiality, including anonymization of identifying details. Consent was provided voluntarily, and the patient was informed of their right to withdraw consent at any time prior to publication without any consequences to their care.

## Data Availability

The original contributions presented in the study are included in the article/Supplementary material, further inquiries can be directed to the corresponding author.
