# Supplementary material for: Severe hypoglycemic coma with radiological evidence of hypoglycemic encephalopathy due to impaired awareness to hypoglycemia: a case report
Source: Front Med (Lausanne). 2026 Mar 11;13:1680929. doi: 10.3389/fmed.2026.1680929 (PMC13015962; doi:10.3389/fmed.2026.1680929)
Supplement: Supplementary file 1 [file Table_1.docx]

# Lab Report Table S1

| Description | Result Value | Reference Range | Result Value Flag |
| --- | --- | --- | --- |
| Albumin serum | 35.0 | 35-50 |  |
| Alkaline Phosphatase | 83 | 40-150 |  |
| Direct Bilirubin | 3.5 | < 8.6 |  |
| Total Bilirubin | 8 | 5.1-20.5 |  |
| Urea | 5.20 | 3.2-7.4 |  |
| NT-proBNP (N-term) | 126.70 | < 125 | H |
| Troponin I HS | 0.118 | < 0.028 | H |
| Chloride | 111 | 98-107 |  |
| Bicarbonate (CO2) | 17 | 22-29 | L |
| Creatinine | 76.9 | 62-104 |  |
| Gamma GT | 45 | 1-64 |  |
| AST/GOT | 45 | < 35 | H |
| ALT/GPT | 58 | < 55 | H |
| CRP High Sensitivity | 327.06 | < 10 | H |
| Potassium | 4.3 | 3.5-5.1 |  |
| LDH | 135 | 125-225 |  |
| Magnesium | 0.81 | 0.66-1.07 |  |
| Sodium | 139 | 136-145 |  |
| Phosphorus | 0.66 | 0.74-1.52 | L |
| Procalcitonin | 0.22 | < 0.5 |  |
| WBCs | 15.19 | 4-11 | H |
| NEUTROPHILS | 12.28 | 2-6.9 | H |
| NEUT% | 80.870 | 37-80 | H |
| LYMPHOCYTES | 1.338 | 1-4 |  |
| LYM% | 8.81 | 10-50 | L |
| MONOCYTES | 1.439 | 0.2-0.9 | H |
| MONO% | 9.47 | 1-11 |  |
| EOSINOPHILS | 0.011 | 0-0.5 |  |
| EO% | 0.071 | 0-5 |  |
| BASOPHILS | 0.118 | < 0.2 |  |
| BASO% | 0.779 | 0-2.5 |  |
| RBCs | 4.31 | 4.04-6.13 |  |
| Hemoglobin | 14.9 | 13-17.4 |  |
| Hematocrit | 43.6 | 39-52 |  |
| MCV | 101.2 | 78-98 |  |

# Additional Lab Report Table S2

| Description | Result Value | Reference Range | Result Value Flag |
| --- | --- | --- | --- |
| LDH | 216 | 135-225 |  |
| Magnesium | 0.81 | 0.66-1.07 |  |
| Sodium | 139 | 136-145 |  |
| Phosphorus | 0.66 | 0.74-1.52 | L |
| Procalcitonin | 0.22 | < 0.5 |  |
| WBCs | 15.19 | 4-11 | H |
| NEUTROPHILS | 12.28 | 2-6.9 | H |
| NEUT% | 80.870 | 37-80 | H |
| LYMPHOCYTES | 1.338 | 0.6-4 |  |
| LYM% | 8.81 | 10-50 | L |
| MONOCYTES | 1.439 | 0.2-0.9 | H |
| MONO% | 9.47 | 1-11 |  |
| EOSINOPHILS | 0.011 | < 0.5 |  |
| EO% | 0.071 | 0-5 |  |
| BASOPHILS | 0.118 | < 0.2 |  |
| BASO% | 0.779 | 0-2.5 |  |
| RBCs | 4.31 | 4.04-6.13 |  |
| Hemoglobin | 14.9 | 13-17.4 |  |
| Hematocrit | 43.6 | 39-52 |  |
| MCV | 101.2 | 78-96 | H |
| MCH | 34.5 | 27-32 | H |
| MCHC | 34.1 | 29-37 |  |
| RDW | 12.7 | 11.6-15.5 |  |
| Platelets | 315 | 150-450 |  |
| MPV | 7.87 | 7.4-10.4 |  |
| PT (Prothrombin Time) | 12.90 |  |  |
| INR (International N.) | 1.08 |  |  |
| PTT (Partial Thromb.) | 28.60 | 25-33 |  |
| D-DIMER | 0.37 | 0-0.5 |  |
| HbA1c (Glycosylated) | 6.90 |  | H |
| Insulin (Random) | 1.16 | 0.35-4.94 |  |
| TSH | 1.148 |  |  |
| C-Peptide | < 0.03 | 1.11-4.43 |  |

# Table S3 showing urine analysis

| Description | Result Value | Reference Range |
| --- | --- | --- |
| Color | LIGHT YELLOW |  |
| Appearance | Clear |  |
| Sp. Gravity | 1.034 | 1.005-1.03 |
| pH | 6.0 | 4.6-8 |
| Nitrites | Nil | Nil |
| Protein | Trace | Nil |
| Glucose | 3+ | Nil |
| Ketones | 3+ | Nil |
| Urobilinogen | NORMAL | Normal |
| Bilirubin | Nil | Nil |
| UWBC | 15-25 | Nil |
| URBC | 5-10 | Nil |
| Bacteria | Nil | Nil |
| Epithelial Cells | Nil | Nil |
| Crystal Urine | Nil | Nil |
| Mucus | Nil | Nil |
| Others | Nil | Nil |
| Leukocytes | 2+ | Nil |
| Blood | 1+ | Nil |
| Ammonium Biurate | Nil | Nil |
| Amorphous Phosphat | Nil | Nil |
| Amorphus Urate Cry. | Nil | Nil |
| Calcium Carbonate | Nil | Nil |
| Calcium Oxalate Cry. | Nil | Nil |
| Cholesterol Crystals | Nil | Nil |
| Cysteine Crystals | Nil | Nil |
| Triple phosphate Cry. | Nil | Nil |
| Tyrosine Crystals | Nil | Nil |
| Uric Acid Crystals | Nil | Nil |
| Granular Casts | Nil | Nil |
| Hyaline Casts | Nil | Nil |
| RBCs Casts | Nil | Nil |
| WBCs Casts | Nil | Nil |
| Waxy Casts | Nil | Nil |
| Yeast Cells | Nil | Nil |

# Table S4 showing serology results

| Description | Result Value | Reference Range |
| --- | --- | --- |
| Granular Casts | Nil | Nil |
| Hyaline Casts | Nil | Nil |
| RBCs Casts | Nil | Nil |
| WBCs Casts | Nil | Nil |
| Waxy Casts | Nil | Nil |
| Yeast Cells | Nil | Nil |
| Schistosoma Ova | Nil | Nil |
| Trichomonas vaginalis | Nil | Nil |
| RBCs Morphology (N) | Nil | Nil |
| RBCs Morphology (D) | Nil | Nil |
| EPS-WBCs | Nil | Nil |
| Sperm Cells | Nil | Nil |
| General Remarks U/A | Nil | Nil |
| Adenovirus (PCR) | Negative | Negative |
| Bocavirus (PCR) | Negative | Negative |
| Coronavirus 229E (PCR) | Negative | Negative |
| Coronavirus HKU1 (PCR) | Negative | Negative |
| Coronavirus NL63 (PCR) | Negative | Negative |
| Coronavirus OC43 (PCR) | Negative | Negative |
| Human Metapneumo | Negative | Negative |
| Influenza A (PCR) | Negative | Negative |
| Influenza A, subtype H1 | Negative | Negative |
| Influenza A, subtype H3 | Negative | Negative |
| Influenza A, subtype H1N1 2009 | Negative | Negative |
| Influenza B (PCR) | Negative | Negative |
| Parainfluenza virus (1) | Negative | Negative |
| Parainfluenza virus (2) | Negative | Negative |
| Parainfluenza virus (3) | Negative | Negative |
| Parainfluenza virus (4) | Negative | Negative |
| Respiratory Syncytia Virus | Negative | Negative |
| Rhinovirus/Enterovirus | Negative | Negative |
| COVID-19 (SARS-CoV-2 PCR) | Negative | Negative |
| Bordetella Pertussis | Negative | Negative |
| Legionella pneumophila | Negative | Negative |
